# Non-linearity of end-systolic pressure–volume relation in afterload increases is caused by an overlay of shortening deactivation and the Frank–Starling mechanism

**DOI:** 10.1038/s41598-021-82791-3

**Published:** 2021-02-08

**Authors:** Moriz A. Habigt, Michelle Krieger, Jonas Gesenhues, Maike Ketelhut, Mare Mechelinck, Marc Hein

**Affiliations:** 1 Anaesthesiology Clinic, University Hospital RWTH Aachen, Pauwelsstr. 30, 52074 Aachen, Germany; 2 Institute of Automatic Control, RWTH Aachen University, Steinbachstr. 54, 52074 Aachen, Germany

**Keywords:** Translational research, Blood flow

## Abstract

The linearity and load insensitivity of the end-systolic pressure–volume-relationship (ESPVR), a parameter that describes the ventricular contractile state, are controversial. We hypothesize that linearity is influenced by a variable overlay of the intrinsic mechanism of autoregulation to afterload (shortening deactivation) and preload (Frank-Starling mechanism). To study the effect of different short-term loading alterations on the shape of the ESPVR, experiments on twenty-four healthy pigs were executed. Preload reductions, afterload increases and preload reductions while the afterload level was increased were performed. The ESPVR was described either by a linear or a bilinear regression through the end-systolic pressure volume (ES-PV) points. Increases in afterload caused a biphasic course of the ES-PV points, which led to a better fit of the bilinear ESPVRs (r^2^ 0.929 linear ESPVR vs. r^2^ 0.96 and 0.943 bilinear ESPVR). ES-PV points of a preload reduction on a normal and augmented afterload level could be well described by a linear regression (r^2^ 0.974 linear ESPVR vs. r^2^ 0.976 and 0.975 bilinear ESPVR). The intercept of the second ESPVR (V0) but not the slope demonstrated a significant linear correlation with the reached afterload level (effective arterial elastance Ea). Thus, the early response to load could be described by the fixed slope of the ESPVR and variable V0, which was determined by the actual afterload. The ESPVR is only apparently nonlinear, as its course over several heartbeats was affected by an overlay of SDA and FSM. These findings could be easily transferred to cardiovascular simulation models to improve their accuracy.

## Introduction

Since the implantation of ventricular assist devices (VADs) has rapidly evolved as an alternative to transplants in terminal heart failure^1^, interest in cardiovascular physiology at the macroscopic scale has experienced a resurgence. Today, cardiovascular simulation models are important tools to evaluate the interaction between a VAD and the native heart^2^ and to develop appropriate control algorithms^3^. These models require an accurate illustration of the interaction between changes in the loading condition of the heart and the cardiac force. In particular, adaption to afterload is crucial, as VADs are less sensitive to increases in vascular resistance, which consequently leads to an unwanted burden on the ventricle^4^. Most models only implement the Frank-Starling mechanism (FSM) and baroreflex to display fast responses of the heart to changes in loading conditions^5^. Differences in in vivo data are particularly visible in responses to afterload and require adaption of contractility and venous return^6^.

A frequently used concept to represent cardiac contractility is the end-systolic pressure volume relationship (ESPVR)^6–11^, which is based on the time-varying elastance (TVE) model presented by Suga et al.^12^. The ESPVR describes a regression through the end-systolic pressure volume (ES-PV) points that are altered by a ventricular volume or load change, and it is characterized by its slope (Ees) and volume axis intercept (V0). The relationship was initially described as linear and independent of the cardiac load. An increased ventricular contractility can be modeled as an increase in the Ees or a decrease in the V0 (leftward shift). Within this concept, an increased end-diastolic volume (Edv) leads to an increased ventricular pressure and stroke volume (SV). This relationship depicts the FSM, which is also known as the “fast force response to load”^13^. Other intrinsic mechanisms that regulate cardiac output, such as the “slow response to load” (Anrep effect)^14^, shortening deactivation (SDA)^15^ or Gregg phenomenon^16^, are not depicted in the current TVE model and have, to our knowledge, not yet been implemented in computer simulation models of the heart. SDA is a less-known autoregulation mechanism of the heart and describes a decrease in ventricular force generated upon a rapid change in the length of myocardial fibers during contraction. The mechanism is evident in the case of a sudden ventricular afterload decrease. Whereas a low SDA increases the cardiac force in response to an increased afterload during the ejection phase within a heartbeat, the FSM leads to an increase in force between several consecutive heartbeats related to an increase in preload. The Gregg phenomenon describes the increased ventricular contractile function in cases of improved coronary blood flow (CBF), which is accompanied by increased myocardial oxygen consumption. The Anrep effect becomes evident 2–3 min following an afterload increase and reverses the initial ventricular dilation by a consecutive increase in ventricular stroke work (SW).

Soon after the introduction of TVE, it became evident that the ESPVR cannot be considered insensitive to load without restrictions, especially under high loading conditions. The load insensitivity is, for example, questioned by Schipper et al.^17,18^, who reported higher Ees values in experiments in which the afterload was increased compared to those in which the preload was reduced. Similar findings were reported by Baan et al.^19^, who showed an inverse correlation between SV and Ees. These findings can be explained by SDA^15^.

Further unsolved questions concern the shape of the ESPVR. Some authors consider the ESPVR to be linear^12,20,21^, whereas others suggest curvilinearity^13,17,22,23^. For example, some authors have stated that a parabolic ESVPR would better reflect the actual conditions, at least with respect to increased ventricular contractility^22,24^. Pironet et al.^25^ replaced the TVE model with a microscopic computer model of sarcomere contraction to improve the load dependency of the ESPVR. The model was then parametrized to depict canine data. In their study, preload interventions resulted in a curvilinear ESPVR and afterload interventions in a linear ESPVR with a higher slope. The authors did not discuss the effects within the context of SDA or the FSM.

As SDA and the FSM play a relevant role in fast cardiac adaptation to changing loading conditions, it is likely that both mechanisms need to be considered to obtain a correct concept of cardiac contractility. Thus, we hypothesize that the nonlinearity of the ESPVR across a wide load range could be described as a temporal overlay of SDA and the FSM and therefore could be subdivided into two phases. The present porcine study was used to describe the differences between time-related responses of the left ventricle to acute changes in preload and afterload using pressure–volume conductance catheters.

## Methods

### Animal model

#### Animals and maintenance of anesthesia

Twenty-four female pigs (German landrace, 47.8 kg ± 3.3 kg body weight (BW)) received intramuscular premedication (4 mg/kg BW azaperone, Elanco Tiergesundheit AG, Basel, Switzerland) and were subsequently anaesthetized (3 mg/kg BW propofol, Hexal AG, Holzkirchen, Germany) for oral intubation. Further anesthesia was maintained by the insufflation of 0.9–1.2 vol% isoflurane and the continuous application of 6–8 µg/kg BW/h fentanyl. Normoventilation was maintained (Cato, Drägerwerk AG, Lübeck). An infusion of a balanced crystalloid solution (Sterofundin Iso Braun, Melsungen, Germany) was administered at a rate of 6–10 ml/kg BW/h. In addition, a balanced colloid solution (Gelafundin Iso Braun) was used to compensate for blood loss or volume deficiency, which was detected by low cardiac output and hypotension. The body temperature was kept constant (38 °C) by an airflow warming blanket.

### Surgical instrumentation

**Figure 1:**
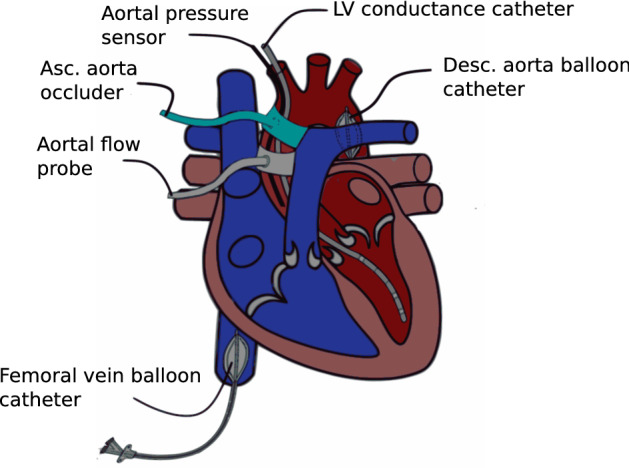
Scheme of the instrumentation of the heart with a conductance catheter, flow and pressure sensors and an inflatable occluder around the ascending aorta and femoral and aortal balloon catheters. (Ao.: aortic, asc.: ascending, desc.: descending, fem.: femoral, LV: left ventricle. Figure is a modification of figure “Heart normal” by Eric Pierce^57^. The original figure can freely be published under CC BY-SA license (https://en.wikipedia.org/wiki/User:Wapcaplet).

One central venous catheter was introduced into the right internal jugular vein, and two 6F sheaths were introduced into the right carotid artery after surgical cut-down. After median thoracotomy, the aorta and pulmonary artery were separated, and a perivascular ultrasound transit-time flow probe (MA 20 PAX; Transonic Systems Europa, Maastricht, the Netherlands) was positioned around the aorta and connected to a flow meter (T402-PV, Transonic Systems Europa). A solid-state pressure sensor (CA-61000-PL, CD Leycom, Zoetemeer, the Netherlands) was introduced through the sheath in the right carotid artery, positioned 3–4 cm distal to the aortic valve and connected to a pressure interface (Sentron, CD Leycom). A multisegment dual-field 7F conductance catheter (SPR-570-7; Millar Instruments, Houston, TX, USA) was inserted through the second right carotid sheath and the aortic valve, with its tip placed in the left ventricular apex. Echocardiographic imaging was performed to verify the correct positioning of the catheter. The catheter was connected to a signal processor (Sigma-5 DF, CD Leycom) and a pressure interface (PCU-2000; Millar Instruments) to obtain the instantaneous left ventricular volume and pressure. Via a 10F sheath in the right femoral vein, an 8F balloon catheter (62080822F, Edwards Lifesciences, Irvine, CA, USA) was placed in the inferior vena cava. Another equal catheter was introduced into the descending aorta via a 10F sheath in the left femoral artery. Finally, an inflatable perivascular occluder was placed distal to the flow sensor around the ascending aorta (vascular occluder 20 mm, In Vivo Metric, Healdsburg, California). Figure 1 presents a schematic overview of the instrumentation. After the instrumentation was completed, the animals recovered for 30 min with continued isoflurane and fentanyl narcosis to achieve stable blood pressure, cardiac output (CO) and normothermia.

### Interventions

**Figure 2:**
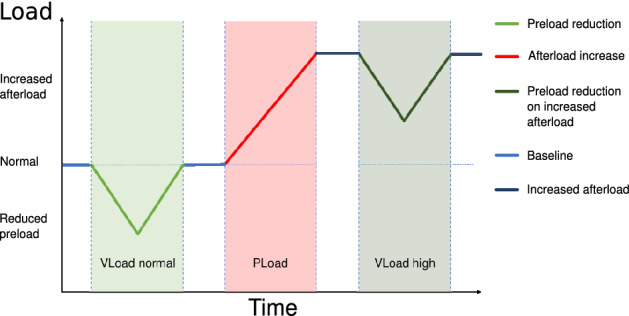
Course of the load interventions with a preload reduction (VLoad normal) followed by an afterload increase (PLoad) and, finally, a preload reduction of an elevated afterload level (VLoad high).

Three different types of interventions were applied: preload reductions (load alteration by volume (V) reduction, VLoad), afterload increases (afterload alteration by aortal pressure (P) increase, PLoad) and preload reductions on an increased afterload (VLoad high). Preload reductions (inferior vena cava occlusion, IVCO) were achieved by shortly (< 10 s) inflating a balloon catheter in the inferior vena cava. Preload reductions were performed on a normal (VLoad normal) and an elevated afterload level (VLoad high). The afterload increases were performed in the ascending aorta and in the descending aorta. These increases were achieved by partial inflation of the occluder around the ascending aorta (ASC) or the intravascular balloon catheter in the descending aorta (DESC). Within every animal, all interventions were conducted with the same balloon catheter position and filling volume, with 3–6 repeats. A new intervention was only performed when the baseline values of the period before the previous intervention had been restored. The experiments were carried out in a random order. A schematic representation of the pre- and afterload interventions is depicted in Fig. 2.

### Data acquisition and calculations

Signals were recorded continuously at a sampling rate of 1000 Hz using a data acquisition device (Powerlab, AD Instruments, Dunedin, New Zealand) and software (LabChart, AD Instruments). The inbuilt cycle detection algorithm LabChart was used to identify single beats using the R-wave of the ECG signal to define the end of diastole. Signals of approximately 20 s in duration around the interventions were exported for further processing with R in RStudio (Version 1.1.456)^26,27^ and several additional packages^28–32^. In this step, extrasystoles were excluded manually. The volume signal was corrected by the parallel conductance calculated from venous hypertonic saline injections and the slope factor α (ratio of SV from conductance signal and aortic flow probe) as described previously^33–35^.

**Figure 3:**
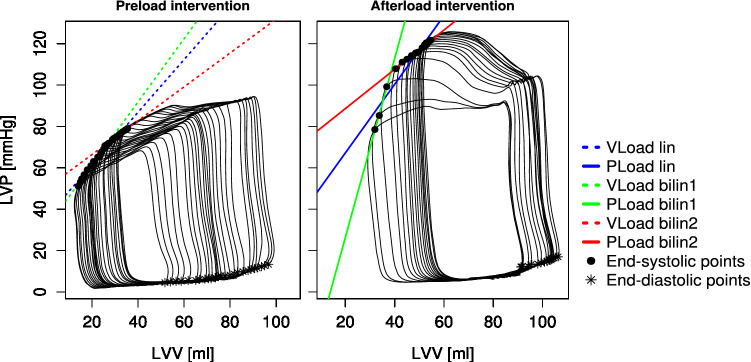
Examples of a pre- and afterload intervention. In both cases, a linear ESPVR (lin) and a bilinear ESPVR consisting of two linear regressions (bilin1 and bilin2) are shown. The end-systolic and end-diastolic points are shown as black dots and stars, respectively. (*LVV* left ventricular volume, *LVP* left ventricular pressure).

The end of systole was set as the maximal elastance point following the equation ${E}_{max}\left(t\right)=\frac{P(t)}{V\left(t\right)-{V}_{d}}$, where the “slack volume” V_d_ was determined iteratively as described by Kono et al.^36^. The ESPVR was calculated only from the section of the intervention where pressure and volume changes occurred. Two different types of regressions were used to describe the ESPVR. A single linear regression through the end-systolic pressure volume points of the complete load intervention with the slope (Ees), x-axis intercept (V0), and coefficient of determination (r^2^) was performed as a marker of the goodness of fit. Furthermore, a “bilinear ESPVR” was determined by selecting the combination of two linear regressions through the end-systolic points, where the geometric mean of the r^2^ values of both regressions was the highest. The intersection of both regression lines defined the end of the first phase (bilin1) and the start of the second phase (bilin2). Exemplary pre- and afterload interventions with the three associated regression lines for the ESPVR are depicted in Fig. 3. The preload recruitable stroke work (PRSW) was described by a single linear regression of the end-diastolic volume and SW. The slope (Mw) and intercept with the volume axis (Vw) reflect ventricular contractility^37^. The following parameters were determined at the start of bilin1 (a), at the end of bilin1 (b), and at the end of bilin2 (c): end-diastolic pressure (Ped) and volume (Ved), end-systolic pressure (Pes) and volume (Ves), SV as the difference of Ved and Ves, SW as the area within the pressure–volume loop, effective arterial elastance (Ea) as the ratio of Pes and SV and the maximal slope of the left ventricular pressure increase (dP/dtmax).

### Statistics

We used SPSS 24.0 software (IBM Corporation, Armonk, USA) to perform the statistical tests. Data are presented as the mean and standard deviation (SD) of the intervention results. A value of p < 0.05 was considered statistically significant. To create the graphics and to visualize the data, paint.net (Rick Brewster, dotPDN, L.L.C., San Francisco, USA), RStudio^27^ and Prism (PRISM 8.1, GraphPad Software, San Diego, USA) were used.

The effect of the intervention type (VLoad, PLoad) on Ees and r^2^ of different ESPVRs was analyzed by two-way variance analysis with Dunnett´s post hoc test for multiple comparisons. Variance analysis for repeated measurements was used to describe the significant effects of the intervention type and location on the ESPVR and PRSW values and on other hemodynamic parameters. A T-test was used for post hoc analysis of the differences between the site of intervention (ASC vs. DESC), and Dunnett´s correction was used for significant changes from the normal loading condition. Finally, we investigated the correlation between different values of Ea, Ees and V0 using Pearson’s correlation coefficient.

### Ethics approval and consent to participate

All procedures described are compliant with the Guide for the Care and Use of Laboratory Animals^55^ and the ARRIVE Guidelines^56^ and have been reviewed and approved by the local animal care committee and the governmental animal care office (No. 84-02.04.2013. A476; Landesamt für Natur-, Umwelt- und Verbraucherschutz Nordrhein-Westfalen, Recklinghausen, Germany).

## Results

A visible nonlinearity of the ESPVR during the afterload interventions (Fig. 3) compared to the preload interventions was observed in all animals.

Accordingly, the mean values of Ees lin, Ees bilin1 and Ees bilin2 were significantly different (P < 0.001) and were dependent on the intervention type (VLoad, PLoad; P < 0.001). The goodness of fit for the simple linear regression (lin) was significantly lower in the afterload interventions than the preload interventions and could be improved by bilinear regression analysis (Table 1). Overall, the differences between the identified Ees values (Ees lin/bilin1/bilin2) were lower for the preload interventions than the afterload interventions. The afterload interventions were characterized by higher Ees values, especially within the first phase (bilin1). Consequently, for further analysis, the ESPVR was described by a single linear regression (lin) for the preload interventions and a bilinear regression (bilin1/bilin2) for the afterload interventions.

**Table 1:** Effect of preload (VLoad) and afterload (PLoad) interventions on the slope (Ees) and goodness of fit (r^2^) of the ESPVR derived from single linear regression (lin) and two phases of bilinear (bilin1, bilin2)) regression.

	Type of regression					
	lin		bilin1		bilin2	
	Mean	SD	Mean	SD	Mean	SD
***Ees***						
VLoad	1.21	0.51	1.44*	1.19	1.06*^#^	0.53
PLoad	2.28^$^	0.80	3.36*^$^	1.49	1.54*^#$^	0.65
***r***^***2***^						
VLoad	0.974	0.021	0.976	0.030	0.975	0.060
PLoad	0.929^$^	0.068	0.960*	0.055	0.943^$^	0.094

The means and standard deviations (SD) are shown (*p < 0.05 vs. lin; ^#^p < 0.05 vs. bilin1, ^$^p < 0.05 vs. VLoad).

**Figure 4:**
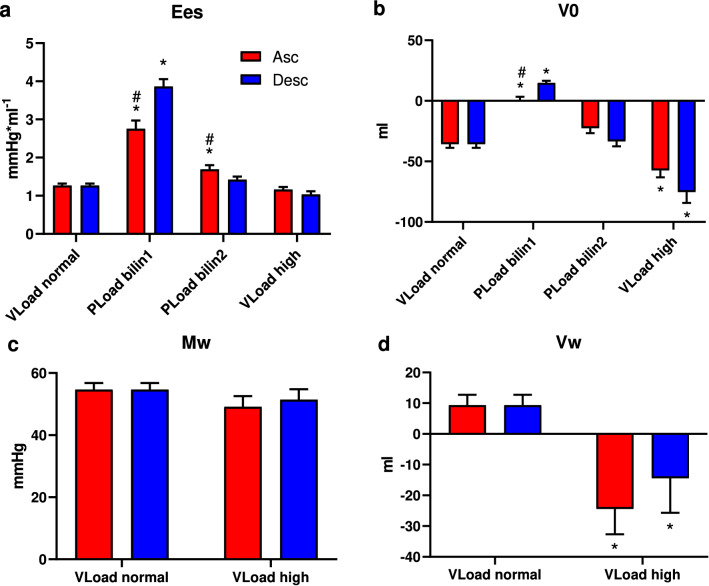
Slope (Ees) and x-axis intercept (V0) of the ESPVR (**a**, **b**) and slope (Mw) and x-axis intercept (Vw) of the PRSW (**c**, **d**) derived from a preload reduction (VLoad normal), the two phases of an increase in afterload (PLoad bilin1 and PLoad bilin2) and a preload reduction of an augmented afterload level (PLoad high). The increases in afterload were achieved by either occlusion of the ascending aorta (ASC) or inflation of a balloon catheter in the descending aorta (DESC). (Mean values ± SD; *p < 0.05 vs. VLoad normal, ^#^p < 0.05 vs. DESC).

Afterload elevation by occlusion of the ascending or descending aorta led to an increase in Ees (Fig. 4a) only in the first phase (lin1) and was associated with a significant increase in V0 in this phase (Fig. 4b). Whereas the Ees values had comparable levels in the second phase of PLoad (bilin2) and for VLoad high, the ESPVR was significantly shifted to the left (decrease in V0) during the VLoad high phase (Fig. 4a/b).

Comparable effects on the PRSW could be found. Mw remained stable (Fig. 4c) but Vw decreased significantly during the VLoad high phase (Fig. 4c). As the PRSW was nearly horizontal or yielded negative values during PLoad, further analysis was not meaningful (data not shown).

**Figure 5:**
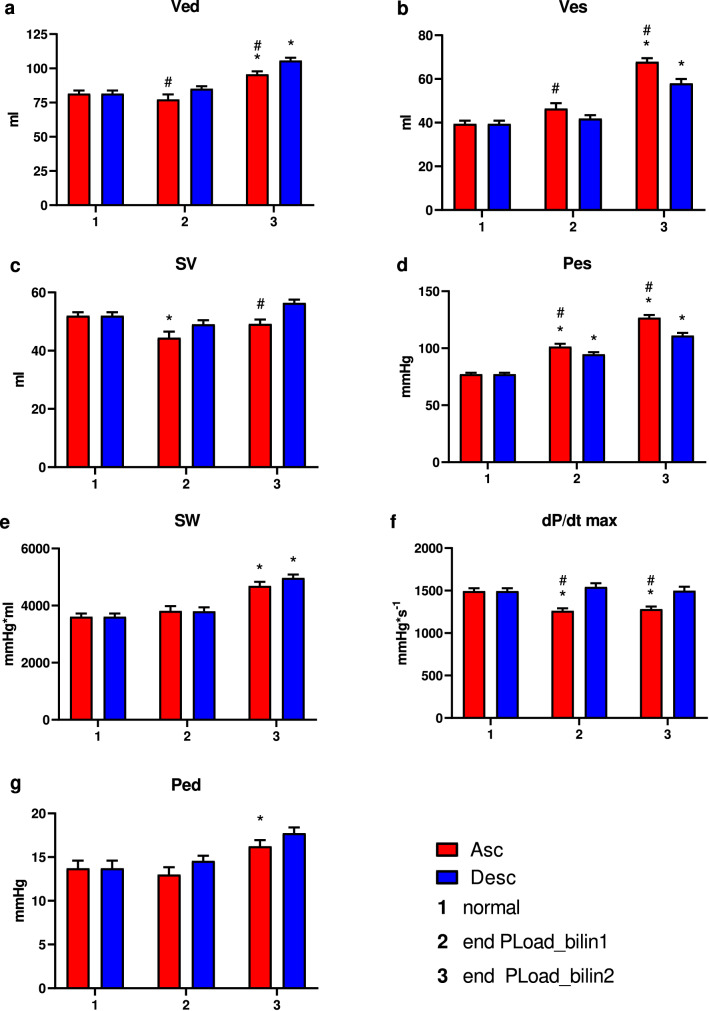
Ventricular parameters under a normal load (a) and at the end of the first (PLoad bilin1) and the second (PLoad bilin2) phases of the afterload interventions by either occlusion of the ascending aorta (ASC) or inflation of a balloon catheter in the descending aorta (DESC). (MW ± SD; *p < 0.05 vs. normal load; ^#^p < 0.05 vs. DESC; Ved = end-diastolic volume, Ves = end-systolic volume, SV = stroke volume, Pes = end-systolic pressure, SW = stroke work, dP/dtmax = maximal rate of intraventricular pressure change, Ped = end-diastolic pressure).

During the first phase of the response to the afterload increase (PLoad bilin1), no increase in Ved, dP/dtmax or SW was observed in our investigation. However, the slope of the resulting ESPVR was nearly two times higher than that observed during the second phase of the response. In contrast, the afterload increase and the consecutive rise in ventricular pressure led to increases in Ved, Ves, SW and Ped at the end of the second phase of the afterload intervention (bilin2, Fig. 5). A decrease in SV could be observed only during the first phase (bilin1) of PLoad. Lower levels of dP/dtmax could be measured only after occlusion of the ascending aorta (ASC). Load interventions in the descending aorta (DESC) led to higher values of Ees bilin1 and V0 bilin1 but lower values of Ees bilin2 compared to ASC interventions. Simultaneously, Ved, SV and dP/dtmax were higher and Ves was lower after occlusion of the descending aorta (DESC) (Fig. 5).

When considering the characteristics of the isovolumic contraction phase on the increased afterload level, a prolonged duration became evident, whereas dP/dtmax and time to dP/dtmax remained unchanged (data not shown).

The intra- and interindividual variability of Ea was useful for further analysis of the effect of the afterload levels on the ESPVR indices. The Ea measured at different phases demonstrated a significant negative correlation only with V0, not with Ees. Pearson’s correlation coefficient was higher in the second phase of PLoad (Table 2). Thus, an acute increase in Ea leads to a leftward shift of the ESPVR.

**Table 2:** Pearson’s correlation (r) between the mean Ees and V0 values and the mean maximally generated elastance values (Ea) before intervention (normal) and at the end of the different afterload intervention phases (Ea PLoad bilin 1 and 2) in all animals.

	Ees bilin1	Ees bilin2		V0 bilin1		V0 bilin2	
**Ea normal**							
*r*	0.065		− 0.152		− 0.547		− 0.579
*p*	0.532		0.143		< 0.001		< 0.001
**Ea PLoad bilin1**							
*r*	− 0.168		− 0.016		− 0.349		− 0.254
*p*	0.140		0.891		0.002		0.025
**Ea PLoad bilin2**							
*r*	0.185		− 0.043		− 0.464		− 0.486
*p*	0.074		0.682		< 0.001		< 0.001

## Discussion

According to the data presented in this study, two phases can be distinguished in the ventricular response to an acute afterload increase. As a result, the regression of the ES-PV points can be well described by a bilinear ESPVR, where the slope of the second phase (Ees PLoad bilin 2) corresponds to a preload intervention and the x-axis intercept V0 with the actual afterload level. Thus, early responses to preload and afterload could be described by a linear ESPVR with a fixed slope and variable intercept. Variable shares of SDA and the FSM on the total effect of the changes in contractility in response to load resulted in only an apparently nonlinear course.

As no dilation of the ventricle occurred in the first phase of the performed afterload increases (PLoad bilin1), the increase in force to generate higher pressure values, which was at the expense of a lower SV, could not be related to the FSM. The forces of the acute afterload increases were transferred to the ventricle in the early ejection phase, where the preload of the ventricle remained unchanged. Consequently, the adaptation that leads to these effects must be triggered in an early phase of the cardiac cycle. The known regulatory mechanisms that might explain these findings are reduced SDA or increased coronary perfusion pressure (Gregg phenomenon). The interrelation between the velocity of ejection and left ventricular pressure has already been described by Suga et al.^38^. They showed that an induced increase in ejection velocity results in reduced pressure generation during ejection which corresponds to the SDA. Thus, the manipulation of the loading condition during ejection, which affects the ejection velocity, also affects the pressure generation and the end-systolic pressure volume relation^39^. These findings are supported by the work of Schotola et al.^40^, who investigated the effects of afterload and preload on force generation in isolated heart muscle preparations of rabbits. Accordingly, the response to preload alterations is modulated by the afterload level. It was postulated that the afterload level increased contractility independent of the FSM, which might be explained by the decreased length-dependent deactivation.

Additionally, it was shown that increases in coronary arterial pressure and coronary blood flow, which may be caused by increased aortic pressure, are associated with improved left ventricular function (Gregg phenomenon)^16,41^. However, these findings, which were shown in isolated hearts^42^ and hearts with extracorporeal circulation^43^, could not be confirmed in porcine experiments^44^. Despite significant changes in CBF within a wide range of perfusion pressures (70–186 mmHg), no changes in contractility could be observed. Thus, the relevance of the Gregg phenomenon for our results seems unlikely.

In our data, the intersection of the two linear regressions (bilin1 and bilin2) and initiation of ventricular dilation occurred at the same time and represented the transition from the first to the second phase of the ventricular response to acute afterload. The second phase of adaption (PLoad bilin2) demonstrated the typical characteristics of the FSM: ventricular dilation, which coincides with an increased Ped and leads to an increased SW^45^. An increased ventricular SW could also be explained by the Anrep effect^14^. However, the usually slower onset of this mechanism and the observed ventricular dilation contradict the relevant role of this effect in the observed phase of the afterload increase.

Other investigators also observed increased but linear ESPVRs during afterload interventions^18,19^. This finding might be explained by a slower increase in load, which took approximately 10–20 s in these studies. In contrast, we increased the load within 1–2 s. The phase in which the high Ees values could be observed, reflecting SDA, lasted for a maximum of 5 s. Increasing the afterload within a shorter period of time also led to a nonlinear ESPVR in other studies^22,46^. In these studies, the ES-PV points were finally described by a parabolic ESPVR, whereas the visually presented ES-PV points could be described by a bilinear ESPVR. Kass et al.^22^, who also stated that the linearity of the ESPVR is dependent on the load range induced on the ventricle and that “nonlinearity may arise from ejection history,” also fitted two linear ESPVRs to each intervention in their investigation. The authors even described the slope of the regression of the second intervention phase as being similar to the slope of a preload intervention, which supports our findings as well. Unfortunately, the concept was not pursued further, as the authors considered the afterload increase as one phase that should be described by only one regression. This point of view might be one reason why the ESPVR resulting from an acute afterload increase has not yet been described as a bilinear regression. Additionally, some authors assume that in the range of high filling pressures, exhaustion of the FSM occurs^47^, and that higher ventricular filling pressures are no longer accompanied by higher ventricular force generation, which can be well described by a parabolic ESPVR. This concept has not been proven and does not play a role in a physiologic filling pressure range^22^, but it might explain why these authors preferred to describe the ESPVR as parabolic.

**Figure 6:**
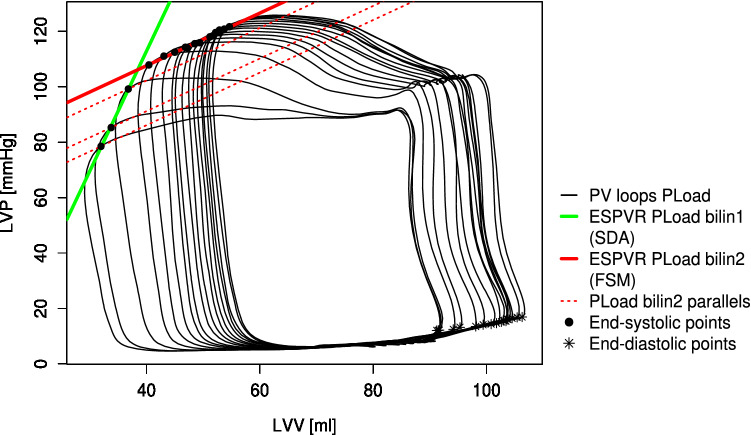
Pressure volume loops of an afterload intervention with the associated end-systolic pressure volume relationships (ESPVR) obtained from the first (PLoad bilin1) and second phases (PLoad bilin2). The end-systolic values (black dots) of PLoad bilin1 are components of different ESPVRs (dotted red lines) with a slope equal to that of the ESPVR bilin2 values. Phase 1 reflects the mechanism of “shortening deactivation”, and phase 2 represents the Frank-Starling mechanism or “fast force response”.

The novelty of the concept presented in this study is the accountability of multiple regulation mechanisms as being responsible for the acute ventricular response to an afterload increase. Within this concept, a subdivision of the ventricular response into two phases and the consecutive description of the single phases by a linear ESPVR seems reasonable. Thus, the first phase of the ESPVR can be described as afterload dependent. This was also stated by other authors^18,46,48^, who described the ESPVR as one linear or a parabolic regression. In contrast to these authors, a load dependency of the ESPVR in the second phase (PLoad bilin2) was not found in our data. Here, only the volume intercept V0 of PLoad bilin2 seems to correlate with the afterload level (Ea), as shown in the results section (Table 2). Within the presented concept, the first phase of the ESPVR (PLoad bilin1) can also be interpreted as a sequence of pressure volume loops (PV-loops) with a constant slope of the (single beat) ESPVR and a variable volume intercept V0 (Fig. 6; dotted red lines). Consequently, the ESPVR resulting from an acute afterload increase can be described by two linear regression lines with different slopes and x-axis intercepts or one ESPVR with a constant slope and an intercept correlated with the actual afterload.

These assumptions are based on the comparability of the Ees values obtained from the VLoad normal, VLoad high and PLoad bilin2 parameters and the correlation of V0 obtained from PLoad bilin2 with the achieved afterload level (Ea).

VADs are less sensitive to afterload, and increases in VADs impair global flow and increase cardiac load^4^. Although no direct comparisons of in vivo and in silico data exist, the observed dilation of the ventricle seemed to be more pronounced in simulation studies^11,49^ than in in vivo studies^50,51^. This observation might confirm the need for an implication of SDA. The linear connection between V0 and Ea facilitates a possible formulation of the interrelation between the afterload and the consecutive leftward shift of the ESPVR (SDA) in a computer simulation algorithm. Only an additional implementation of an inverse correlation between V0 and the afterload (Ea) needs to be implemented, as it could be averaged by our data: $V0 = -24.2 x Ea + 23$.

**Figure 7:**
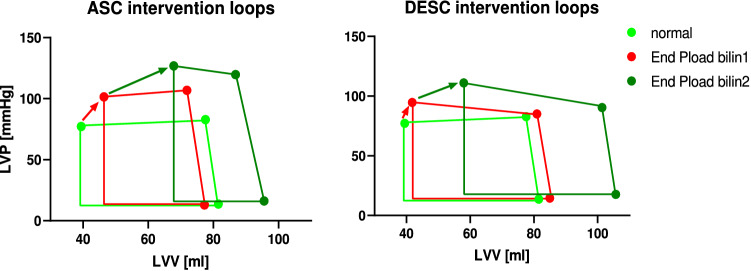
Mean volume and pressure values of all animals before intervention (normal), at the end of the first phase of the pressure intervention (End PLoad bilin1) and at the end of the second phase of the pressure intervention (PLoad bilin2). The results for the ascending aorta interventions (ASC) and for the descending aorta intervention (DESC) are shown separately.

Clearly, the type of afterload intervention (ASC vs. DESC) significantly influences the ventricular behavior during the intervention. Although occlusion of the ascending aorta (ASC) led to a higher increase in pressure, occlusion of the descending aorta (DESC) led to more pronounced ventricular dilation (Fig. 7). These effects probably resulted from the different effects of the interventions on baroreflex and aortic compliance. Whereas occlusion of the descending aorta (DESC) increased the pressure in the carotid artery, occlusion of the ascending aorta (ASC) induced a pressure decrease in the carotid artery. The resulting activation of the baroreflex during an intervention of the descending aorta (DESC) counteracted the increase in contractility (lower Ees during PLoad bilin2, Fig. 4), which led to increased ventricular dilation. This effect was previously described by Oikawa et al.^52^. Furthermore, occlusion of the ascending aorta (ASC) led to a larger decrease in ventricular compliance compared to the compliance observed upon occlusion of the descending aorta, which resulted in increased afterload levels. Kelly et al.^53^ observed a trend toward higher values for indices of contractility after an isolated reduction of central compliance. The authors stated that the history of ejection might affect cardiac function.

An important limitation of our study is the missing control of the afterload levels. A prior definition of several stages could have improved the findings. For a further analysis of SDA, time-related control of the afterload during the early or late ejection phase is necessary. Additionally, it has been shown that myocardial afterload dependency is less pronounced in short-term hibernation than in the ischemic and reperfused myocardium^54^. How acute ischemia and reperfusion effects affect the findings of our study needs to be clarified in further investigations.

## Conclusion

An acute afterload increase results in a two-phased ventricular response in our experiments, reflecting SDA and the FSM successively in time. The resulting ESPVR could be described by two linear regression lines with different slopes an x-axis intercepts or one ESPVR with a constant slope and an intercept correlated with the actual afterload. This leftward shift will lead to an increase in contractility and thus less dilation (preload) of the ventricle. This effect will affect and possibly improve cardiovascular simulation models, which were used to describe the interaction of the heart and the VAD and develop load-sensitive controllers.

## Data Availability

The datasets used and analyzed during the current study are available from the corresponding author on reasonable request.
